# Development of Intron Polymorphism Markers and Their Association With Fatty Acid Component Variation in Oil Palm

**DOI:** 10.3389/fpls.2022.885418

**Published:** 2022-06-02

**Authors:** Jing Li, Yaodong Yang, Xiwei Sun, Rui Liu, Wei Xia, Peng Shi, Lixia Zhou, Yong Wang, Yi Wu, Xintao Lei, Yong Xiao

**Affiliations:** ^1^Hainan Key Laboratory of Tropical Oil Crops Biology/Coconut Research Institute, Chinese Academy of Tropical Agricultural Sciences, Wenchang, China; ^2^Institute of Tropical Agriculture and Forestry, Hainan University, Haikou, China; ^3^Tropical Crops Genetic Resources Institute, Chinese Academy of Tropical Agricultural Sciences, Haikou, China

**Keywords:** *Elaeis guineensis*, fatty acid, IP markers, candidate gene, association analysis

## Abstract

Oil palm (*Elaeis guineensis* Jacq.) is a tropical woody oil crop of the palm family and is known as “the oil king of the world,” but its palm oil contains about 50% palmitic acid, which is considered unhealthy for humans. Intron polymorphisms (IP) are highly efficient and easily examined molecular markers located adjacent to exon regions of functional genes, thus may be associated with targeted trait variation. In order to speed up the breeding of oil palm fatty acid composition, the current study identified a total of 310 introns located within 52 candidate genes involved in fatty acid biosynthesis in the oil palm genome. Based on the intron sequences, 205 primer pairs were designed, 64 of which showed polymorphism among 70 oil palm individuals. Phenotypic variation of fatty acid content in the 70 oil palm individuals was also investigated. Association analysis revealed that 13 IP markers were significantly associated with fatty acid content variation, and these IP markers were located on chromosomes 2, 5, 6, 8, 9, and 10 of oil palm. The development of such IP markers may be useful for the genetic improvement of fatty acid composition in oil palm.

## Introduction

Oil palm (*Elaeis guineensis*, 2n = 32) is an important tropical oil crop and is often referred to as “the oil king of the world” because it has the highest oil yield per unit area among all oil crops (Singh et al., 2013; Huang, 2017). Global production of palm oil in 2017 was approximately 75.70 million tons (Mozzon et al., 2020). The two oil storage tissues of oil palm are the mesocarp and kernel, each of which produces oil with a different fatty acid composition. Palmitic acid (16:0) is the major fatty acid (about 50%) in oil from the oil palm mesocarp, while lauric acid (12:0) is the major fatty acid (about 50%) in kernel oil. As for many important oil crops, improving fatty acid content is a major breeding objective for this tropical oil crop, especially aiming to decrease the palmitic acid content and increase the oleic acid content. However, the breeding scheme is slow due to the long life cycle of oil palms. Developing molecular markers associated with fatty acid compositions could facilitate the breeding and selection of the tropical oil-seed crop.

Molecular markers such as amplified fragment length polymorphisms (AFLPs), random amplified polymorphic DNA (RAPD), and restriction fragment length polymorphisms (RFLPs) have been widely used for analyzing genetic diversity and population structure, identification of trait-associated markers, and genotype characterization in oil palm (Moretzsohn et al., 2000; Rance et al., 2001; Billotte et al., 2005; Cochard et al., 2009; Singh et al., 2009; Ting et al., 2013). In eukaryotic genomes, there are generally some introns across each gene (Hawkins, 1988; Deutsch and Long, 1999; Haas et al., 2005). Diversity of intron sequences between different individuals is abundant due to low selection pressure (Stoltzfus et al., 1997; Panjabi et al., 2008; Sharma et al., 2020). Therefore, numerous intron polymorphism (IP) markers are available in the eukaryotic genome (Badoni et al., 2016; Kita et al., 2016). Studies have identified different types of IP, including ILP (Intron Length Polymorphism; Wang et al., 2005; Sharma et al., 2020) and ISNP (Intron Single Nucleotide Polymorphism; Ferreira et al., 2009; Liu et al., 2018). IP markers are generally co-dominant and highly polymorphic, and are widely used for constructing genetic maps, diversity analysis, and quantitative trait locus mapping (Williams et al., 1990; Wei et al., 2005; Yang et al., 2007; Xia et al., 2017). In 2013, the genome sequence of oil palm was released, providing an opportunity to develop larger numbers of IP markers (Singh et al., 2013). Polymorphism markers located in the intron region can be efficient function markers in genic regions. Therefore, IP in targeted genes may be associated with targeted traits in *E. guineensis*.

In this study, candidate genes and their introns involved in the biosynthesis and metabolism of fatty acids were identified in the genome sequence of *E. guineensis*. Subsequently, IP markers were developed based on intron sequences among 70 oil palm individuals. Fatty acid composition was also investigated among the 70 oil palm individuals. Finally, associations between IP markers and fatty acid variation were analyzed. This study will provide a exhaustive understanding of fatty acid content in oil palm, and the IP makers and candidate genes detected will facilitate breeding for fatty acid content in oil palm. Provide a theoretical basis for increasing the content of oleic acid and reducing the content of palmitic acid in future oil palm breeding.

## Materials and Methods

### Plant Materials and DNA Extraction

A total of 70 oil palm individuals were selected from the oil palm germplasm resources of the Coconut Research Institute of Chinese Academy of Tropical Agricultural Sciences, Wenchang town, Hainan province, China. Detailed information for these oil palm individuals is listed in Supplementary Table 1. DNA samples were prepared from spear leaves using the mini-CTAB method (Murray and Thompson, 1980).

### Extraction and Measurement of Fatty Acid Contents in Oil Palm Mesocarp Tissues

Three fruits per oil palm individual (three biological replicates) were harvested, and fatty acid extraction and analyses for each mesocarp tissue were performed in triplicate (three different

extractions as technical replicates). Approximately 60 mg mesocarp was used for extracting fatty acids according to methods described by Li-Beisson et al. (2013). Fatty acid composition was subsequently examined and measured using 7890A gas chromatograph equipped with a HP-5MS column (30 m by 250 μm, 0.25 μm). The heating procedure was as follows: initial temperature 180°C, followed by a temperature increase to 220°C at a rate of 10°C per min. The contents of decanoic acid (C10:0), lauric acid (12:0), myristic acid (C14:0), tripalmitelaidin acid (16:1), palmitic acid (16:0), stearic acid (18:0), oleic acid (18:1), and linoleic acid (18:2) were determined with reference to an internal standard using the following calculation: (1) total fatty acid = (total area of all measured fatty acid peaks × quantity of heptadecanoic acid-methyl ester)/(peak area of heptadecanoic acid-methyl ester × quantity of the sample); (2) relative fatty acid percentage = peak area of a specific fatty acid /total area of all measured fatty acid peaks. The peak area was calculated using Agilent software.

### Identification of Candidate Genes Involved in Fatty Acid Biosynthesis

The whole-genome sequence of *E. guineensis* was downloaded from the National Center for Biotechnology Information (NCBI). Protein sequences involved in fatty acid biosynthesis were downloaded from the Arabidopsis Information Resource (TAIR), available at http://www.arabidopsis.org. The protein sequences from Arabidopsis were used as queries for BLASTx searches against the coding sequence (CDS) database from *E. guineensis* to identify candidate genes involved in fatty acid biosynthesis. Conserved motifs were also predicted by alignment with Conserved Domains Database (CDD)^1^ and PFAM databases.^2^

### Identification of Intron Sequence and Primer Design

CDSs of candidate genes involving in fatty acid biosynthesis were used as queries for BLASTn searches against the genome sequence of *E. guineensis* to identify the boundary between exon and intron. The MEME program^3^ was used to identify the gene structures of candidate genes. Primers were designed based on the intron sequences using Primer3 software (Rozen and Skaletsky, 2000). The list of PCR primers used in this study is provided in Supplementary Table 2.

### PCR Amplification and Electrophoresis

PCR amplification was performed in 10 μl reaction mixtures containing 100 ng genomic DNA, 1× PCR buffer, 2 mM MgCl_2_, 1 U Taq DNA polymerase (TaKaRa, China), 0.5 μM of each primer, and 0.2 mM dNTP mix. The PCR program comprised a denaturation step for 4 min at 94°C, followed by 30 cycles of 30 s at 94°C, 30 s at 54.7°C, and 30 s at 72°C. PCR products were electrophoretically separated on 8% polyacrylamide denaturing gels and visualized by silver staining. Product sizes were determined by comparison with a 100 bp DNA ladder.

### Population Structure and Genetic Diversity Analysis

Bayesian clustering was employed to analyze the population structure of 70 oil palm individuals using the software STRUCTURE (Pritchard et al., 2000). Ten independent calculations were performed for K value (K set from 2 to 7). Burn-in time and replication number were both set to 100,000 in each run. The maximum-likelihood method was applied to assign every individual to a cluster, and the cut-off probability was set to 0.6. The most probable number of populations (K) was identified by plotting ΔK values of K from 1 to 10 in replicate runs for each K and corresponded to the peak of the ΔK graph. Association analysis was conducted using the software Tassel^4^; statistical significance (value of *p*) was determined by 100,000 permutations (Bradbury et al., 2007).

## Results

### Identification of Candidate Genes Involved in *de novo* Synthesis of Fatty Acids

**Figure 1:**
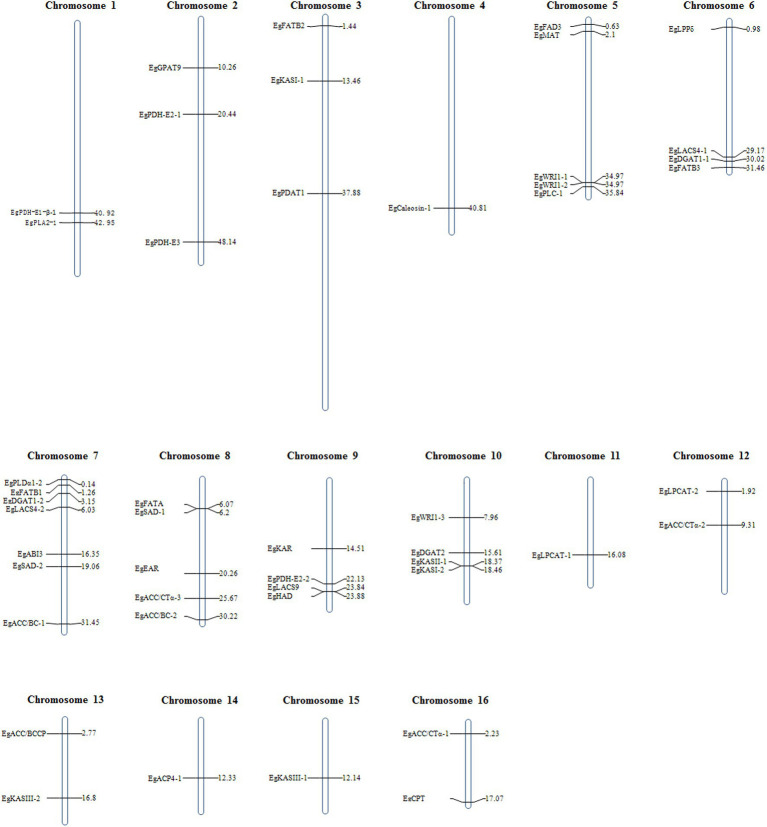
Chromosomal positon of candidate genes involved in the biosynthesis of fatty acid identified in the genome of *E. guineensis.*

The protein sequences involved in *de novo* synthesis of fatty acids from *Arabidopsis thaliana* were used as queries to align against the protein database of *E. guineensis*. A total of 52 candidate genes that might participate in the *de novo* synthesis of fatty acids, carbon termination or dehydration were identified. These candidate genes were distributed across 16 different chromosomes. Among them, the maximum number of candidate genes were detected in chromosome 7, followed by chromosome 5, and then chromosome 9. Chromosomes 4 and 11 each contained only one candidate gene involved in fatty acid biosynthesis (Figure 1).

**Figure 2:**
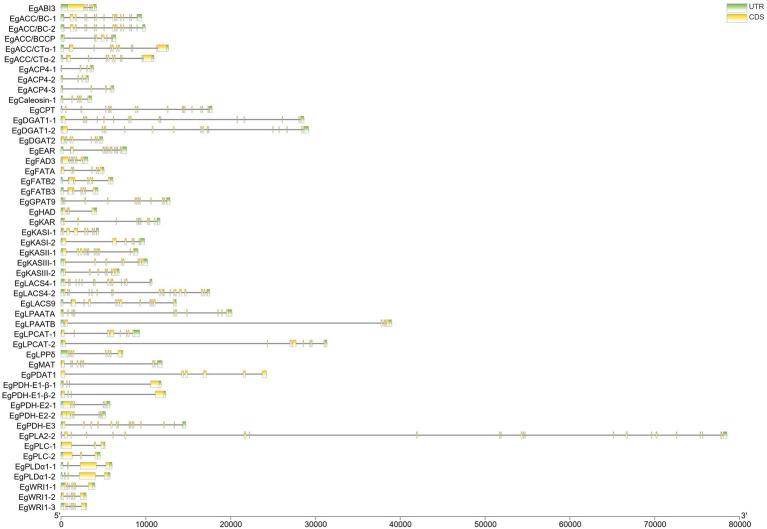
The gene structure of enzyme genes related to the biosynthesis of fatty acid in *Elaeis guineensis.*

CDSs of 52 candidate genes involved in fatty acid biosynthesis were used as queries to align with the *E. guineensis* genome to identify the sequence boundary between intron and exon. Almost all candidate genes contained at least two intron sequences. Among all the candidate genes, the *EgPLA2-2* gene contained the maximum number of introns (22). A large proportion of candidate genes contained approximately 6–7 introns (Figure 2).

### Development of IP Markers Based on the Intron Region of Candidate Genes

**Figure 3:**
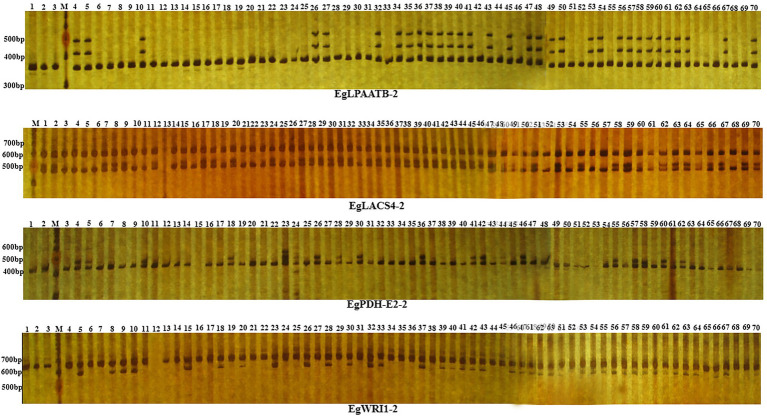
PCR products of IP marker EgLPAATB-2, EgLACS4-2, EgPDH-E2-2 and EgWRI1-2 in 70 oil palm samlpes separated by electrophoresis on 6% non-denaturing PAGE. Lanes 1–70 represent the 70 oil palm samples. M represents 100 bp ladder.

A total of 205 primer pairs were designed based on intron region of candidate genes and were used to amplify the DNA templates of different 70 oil palm individuals (Figure 3). Amplification products from 64 primer pairs showed polymorphisms between oil palm individuals; the observed heterozygosity varied from 0.0286 to 0.9714, with an average of 0.4514. Meanwhile, a total of 81 alleles were identified, with an average of 2.7 alleles per locus. Sizes of the amplification products varied from 120 bp to 1,300 bp, with an average of 424 bp per PCR product. The 81 alleles were located on the intron region of 18 candidate genes involved in fatty acid biosynthesis, were *ACP4*, *CPT*, *EAR*, *FAD3*, *KASII-1*, *LACS4-1*, *LACS4-2*, *LPAATA*, *DGAT1-1*, *PDH-E2*, *PDH-E1*, *WRI1*, *WRI2*, *FatA*, *LACS9*, *LACS4-2*, *LPCAT-1*, and *LPAATB* (Table 1).

**Table 1:** Polymorphism information of IP markers developed in the study.

IP marker	Observed allele	Observed heterozygosity	Shannon diversity index
EgACP4-2	3	0.1	0.2727
EgCPT-1	3	0.3143	0.9594
EgCPT-2	3	0.0286	0.9314
EgEAR	3	0.7286	1.0333
EgFAD3-1	3	0.5429	1.0783
EgFAD3-2	2	0.6143	0.6634
EgKASII-1	4	0.0429	0.9785
EgLACS4-1	3	0.3	0.8568
EgLACS4-2-1	2	0.8	0.6895
EgLACS4-2-2	2	0.6286	0.6332
EgLPAATA	3	0.4571	1.0063
EgDGAT1-1	3	0.1	0.7405
EgFAD3-1	3	0.6857	1.0978
EgFAD3-2	2	0.8429	0.6807
EgPDH-E2-1	3	0.0714	0.3068
EgPDH-E1-1-1	2	0.9	0.6881
EgPDH-E1-1-2	2	0.1	0.6915
EgPDH-E2-2	4	0.2571	0.537
EgPDH-E2-2-1	3	0.4429	1.0061
EgPDH-E2-2-2	2	0.8429	0.6807
EgWRI1-1-1	3	0.3	0.9625
EgWRI1-1-2	2	0.1	0.6895
EgWRI1-2	3	0.5286	0.6479
EgFATA	3	0.9	0.9005
EgLPAATA-1	2	0.1	0.6429
EgLPAATA-2	3	0.2143	0.8535
EgLACS9	2	0.9714	0.6927
EgLACS4-2	3	0.8571	0.7497
EgLPCAT-1	3	0.4571	1.0063
EgLPAATB	2	0.3143	0.4349

### Analysis on the Variation of Fatty Acid Content in 70 Oil Palm Individuals

**Figure 4:**
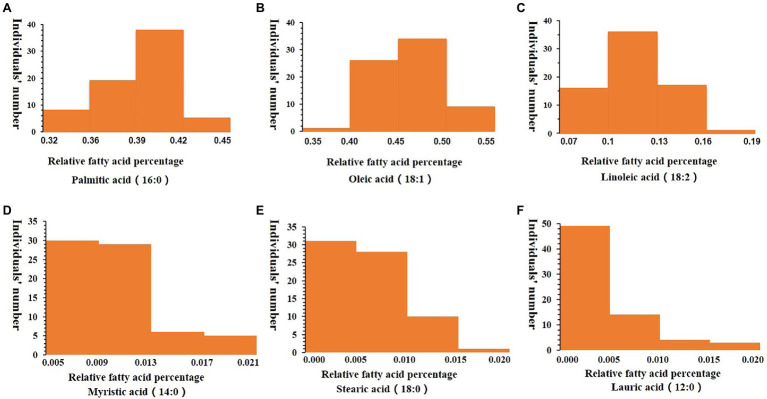
Frequency distribution of relative fatty acid percentages in the mesocarp of 70 oil palm individuals, including palmitic acid (16:0; **A**), oleic acid (18:1; **B**), linoleic acid (18:2; **C**), Myristic acid (14:0; **D**), Stearic acid (18:0; **E**) and Lauric acid (12:0; **F**). The x-axis represents the trait value, and the y-axis represents the number of oil palm individuals.

Fatty acid components of the 70 oil palm individuals were analyzed. Myristic acid content varied from 0.54 to 1.99%, with an average of 1.03%; palmitic acid content varied from 32.7 to 44.32%, with an average of 39.21%; oleic acid content varied from 35.86 to 53.42%, with an average of 46.13%; linoleic acid content varied from 7.17 to 18.07%, with an average of 11.61%; stearic acid content varied from 0.24 to 1.26%, with an average of 0.65%, and lauric acid content varied from 0.03 to 1.97%, with an average of 0.44% (Table 2; Figure 4).

**Table 2:** Statistical values of different fatty acid components in the population of *Elaeis guineensis.*

Statistical values	Myristic acid	Palmitic acid	Oleic acid	Linoleic acid	Steric acid	Lauric acid
Maximum value	1.99%	44.32%	53.42%	18.07%	1.26%	1.97%
Minimum value	0.54%	32.70%	35.86%	7.17%	0.24%	0.03%
Average value	1.03%	39.21%	46.13%	11.61%	0.65%	0.44%
Standard deviation	0.33	2.44	3.08	2.12	0.33	0.43
Variation coefficient	0.32	0.06	0.06	0.18	0.52	0.97

The relationships between different fatty acid components were analyzed using SPSS software. Oleic acid content was negatively correlated with myristic acid content (*r* = −0.408^**^ and *p* = 0.00). Meanwhile, palmitic acid content showed significant negative correlation with oleic acid content (*r* = −0.53^**^ and *p* = 0.00), indicating that decreasing palmitic acid content in oil palm may enhance oleic acid content. Palmitic acid content and oleic acid content both showed significant negative correlation with linoleic acid content (*r* = −0.25^*^ and *p* = 0.04, and *r* = −0.549^**^ and *p* = 0.00 for palmitic acid and oleic acid, respectively). Oleic acid content showed significant negative correlation with stearic acid content (*r* = −0.369^**^ and *p* = 0.002), while linoleic acid content showed significant positive correlation with stearic acid content (*r* = 0.345^**^ and *p* = 0.003; Table 3).

**Table 3:** Association between different fatty acid components.

	Myristic acid	Palmitic acid	Oleic acid	Linoleic acid	Stearic acid
Myristic acid	1	0.12	−0.408^**^	0.05	0.14
Palmitic acid		1	−0.53^**^	−0.25^*^	−0.086
Oleic acid			1	−0.549^**^	−0.369^**^
Linoleic acid				1	0.345^**^
Stearic acid					1

* and ** represent significant at 5% and 1% probability levels respectively.

### Association Between IP Markers and Fatty Acid Components

**Figure 5:**
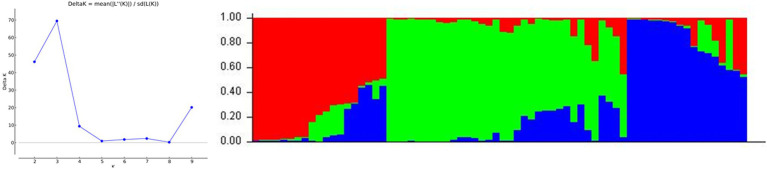
Inferred population structure of 70 oil palm germplasm. Each oil palm individual is represented by a single vertical line. Each color represents one culster. The length of the colored segment indicates the proportion of an individual assigned into one genetic group. The left diagram indicates the true number of genetic clusters.

The developed IP markers were used to evaluate the population structure of 70 individuals of *E. guineensis.* When the method of Evanno et al. (2005) was applied to identify the most likely number of ‘true population’, *K* = 3 genetic groups were found. The STRUCTURE assignment procedure revealed that the largest genetic group comprised 34 oil palm individuals, the second group comprised 19 oil palm individuals, and the remaining group only included 17 oil palm individuals (Figure 5).

A simple linear model (SLM) was used to identify the association between IP markers and fatty acid components. Five IP markers were significantly associated with the variation in myristic acid content, including EgLACS9-2 (*p* = 0.0356 and *r*^2^ = 0.0643), EgPDH-E2-1-3 (*p* = 0.0193 and *r*^2^ = 0.079), EgLACS4-2-3 (*p* = 0.0103 and *r*^2^ = 0.0943), EgWRI1-1-2 (*p* = 0.0475 and *r*^2^ = 0.0574) and EgLPAATB-2 (*p* = 0.0329 and *r*^2^ = 0.0662). Three IP markers showed significant association with linoleic acid, including EgLACS4-1-2 (*p* = 0.0386 and *r*^2^ = 0.053), EgPDH-E2-2-2 (*p* = 0.0286 and *r*^2^ = 0.0591), and EgFATA-1(*p* = 0.0108 and *r*^2^ = 0.0791). One IP marker, EgPDH-E2-2, showed significant association with stearic acid content (*p* = 0.0237 and *r*^2^ = 0.0726). Furthermore, four IP markers were significantly associated with palmitic acid content, including EgPDH-E2-2 -4 (*p* = 0.0134 and *r*^2^ = 0.0856), EgPDH-E2-2 -5 (*p* = 0.0261 and *r*^2^ = 0.0699), EgKASII-1-2 (*p* = 0.0242 and *r*^2^ = 0.0717), and EgKASII-1-3 (*p* = 0.0493 and *r*^2^ = 0.055; Table 4).

**Table 4:** Association between IP markers and fatty acid components.

Trait	Locus	Position	Chromosome	Value of *p*	*R*^2^ (%)
Myristic acid	EgLACS4-2-3	6,030,000	7	0.0103	0.0943
Myristic acid	EgPDH-E2-1-3	20,440,000	2	0.0193	0.079
Myristic acid	EgWRI1-1-2	34,970,000	5	0.0475	0.0574
Myristic acid	EgLACS9-2		–	0.0356	0.0643
Myristic acid	EgLPAATB-2	–	–	0.0329	0.0662
Palmitic acid	EgKASII-1-3	18,370,000	10	0.0493	0.055
Palmitic acid	EgPDH-E2-2-4	22,130,000	9	0.0134	0.0856
Palmitic acid	EgPDH-E2-2-5	22,130,000	9	0.0261	0.0699
Palmitic acid	EgKASII-1-2	18,370,000	10	0.0242	0.0717
Linoleic acid	EgLACS4-1-2	29,170,000	6	0.0386	0.053
Linoleic acid	EgPDH-E2-2-2	22,130,000	9	0.0286	0.0591
Linoleic acid	EgFATA-1	6,070,000	8	0.0108	0.0791
Stearic acid	EgPDH-E2-2-3	22,130,000	9	0.0237	0.0726

### Expression Analysis of Candidate Genes

**Figure 6:**
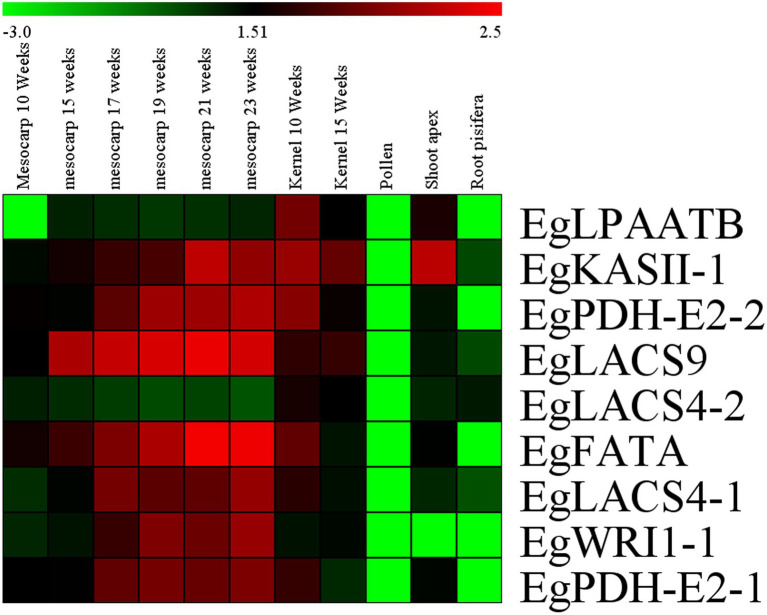
Heat map of candidate genes expression in different tissues of *Elaeis guineensis*. Log_10_^RPKM^ value were sued to construct the heat map with clustering.

To analyze the expression patterns of the candidate genes in different tissues, transcriptomic raw reads data were downloaded from the SRA (Short Read Archive) database of the NCBI website, including SSR851069 (mesocarp 10 weeks after anthesis), SRR190698 (mesocarp 15 weeks after anthesis), SRR190699 (mesocarp 17 weeks after anthesis), SRR190700 (mesocarp 19 weeks after anthesis), SRR190701 (mesocarp 21 weeks after anthesis), SRR190702 (mesocarp 23 weeks after anthesis), SSR851068 (kernel 10 weeks after anthesis), SSR851068 (kernel 15 weeks after anthesis), SSR851099 (pollen), SRR851103 (shoot), and SRR851110 (root). Results of the analysis revealed that almost all candidate genes had a high level of expression in mesocarp and kernel tissues compared with other tissues, except for the gene *EgLACS4-2*. Among them, *EgLPAATB* had higher expression in oil palm kernel compared with that in other tissues. However, seven other candidate genes had a higher expression level in mesocarp and kernel compared with the expression in three other tissues; these genes were *EgKASII-1*, *EgPDH-E2-2*, *EgLACS9*, *EgFATA*, *Eglacs4-1*, *EgWRI1-1*, and *EgPDH-E2-1* (Figure 6).

## Discussion

This study generated a total of 64 polymorphic IP markers, based on intron sequences of candidate genes involved in fatty acid biosynthesis, for use in molecular breeding of oil palm. These markers were screened across 70 oil palm individuals and are predicted to be suitable for widespread use in oil palm breeding, particularly association analysis. This premise was validated by identifying 13 IP markers linked to different fatty acid compositions; these IP markers will be used immediately for marker-assisted selection.

Molecular markers such as simple sequence repeat (SSR), RAPDs, and AFLPs have been widely used for analyzing genetic diversity and population structure, identification of trait-associated markers, and genotype characterization in *E. guineensis* (Moretzsohn et al., 2000; Rance et al., 2001; Billotte et al., 2005; Cochard et al., 2009; Seng et al., 2011; Premkrishnan and Arunachalam, 2012; Myint et al., 2021). The release of the whole genome sequence of *E. guineensis* has provided an opportunity to develop IP markers with regards to targeted candidate genes. Among the various molecular markers, identifying RFLP markers in a genetically diverse population can be cumbersome as it is mainly based on molecular hybridization (Maizura et al., 2006). Moreover, AFLP markers are based on enzyme digestion and PCR amplification with adapter primers and random primers, which is generally unstable (Barcelos et al., 2002). In recent years, a large number of SNP markers have been identified by using next-generation sequencing technology. Xia et al. (2019) revealed 62 SNP markers that were signifcantly associated with fatty acid content, including palmitic acid content (32 SNPs), oleic acid content (4 SNPs), linoleic acid content (1 SNP), and total oil content (25 SNPs) in oil palm. However, SNP remains costly and consequently limits the broad applications of SNP markers for oil palm improvement using these strategies (Ithnin et al., 2021). In contrast, the polymorphisms obtained using IP markers were easily discernable on simple 1.5–2.0% agarose gels and this would enable rapid screening of a large diverse population. In this study, 64 IP markers were developed based on the intron sequences of candidate genes involved in fatty acid biosynthesis, 28.44% of which were polymorphic among different individuals in accordance with previous results (Xia et al., 2019). Moreover, among these IP markers, 20.31% were significantly associated with different fatty acid compositions, including EgLACS4-2-3, EgPDH-E2-1-3, EgWRI1-1-2, EgLACS9-2, EgLPAATB-2, EgFATA-1. The markers EgPDH-E2-2-4, EgPDH-E2-2-5, EgKASII-1-2, and EgKASII-1-3 were significantly associated with variation in palmitic acid content and located in the intron regions of different EgKASII genes that have an important role in carbon extension. Furthermore, EgLACS4-1-2, EgPDH-E2-2-2, and EgFATA-1 markers significantly associated with linoleic acid and located on the *FatA* gene, which is involved in carbon termination of unsaturated fatty acids.

In past several decades, several studies had been performed to identify and validate genes involved in fatty acid biosynthesis. For example, seed-specific RNAi-mediated down-regulation of KASII led to in dramatic increase of palmitic acid (Liu et al., 2017). Our research also showed the IP markers derived from EgKASII-1-2 and EgKASII-1-3 had significant association with palmitic acid composition (*p* = 0.0242 and 0.0493). Meanwhile, our study also indicated that the IP markers located on EgLACS4-1-2 were significantly associated with linoleic acid (*p* = 0.0386 and *r*^2^ = 0.053). LACS has been validated to play a role in linoleic acid biosynthesis in a previous study (Jang et al., 2015). Our results also demonstrated that IP markers obtained from EgWRI1-4 and EgLPAAT were significantly associated with myristic acid. In previous studies, LPAAT catalyzed 14:0-acyl-carrier protein specifically and resulted in high myristic acid content in Cyanothece. However, no documents showed the relationship between EgWRI1-4 and myristic acid.

Therefore, it is possible that some IP markers are closely linked with targeted genes that govern fatty acid content and subsequently show significant associations with targeted traits. In future, these candidate markers could be ideal targets for further study and may have potential application in marker-assisted selection for fatty acid composition.

## Data Availability Statement

The original contributions presented in the study are included in the article/Supplementary Material, further inquiries can be directed to the corresponding authors.

## Author Contributions

YX and WX participated in the design of the study. JL and YY performed the statistical analysis. JL and PS conducted the major experimental work including the extraction and measurement of oil content and relative fatty acid contents. YX and XL wrote the first draft of the manuscript. YWa, YWu, RL, LZ, and XS wrote sections of the manuscript. All authors read and approved the final manuscript.

## Funding

This work was supported by the National Natural Science Foundation of China (no. 31870670).

## Conflict of Interest

The authors declare that the research was conducted in the absence of any commercial or financial relationships that could be construed as a potential conflict of interest.

## Publisher’s Note

All claims expressed in this article are solely those of the authors and do not necessarily represent those of their affiliated organizations, or those of the publisher, the editors and the reviewers. Any product that may be evaluated in this article, or claim that may be made by its manufacturer, is not guaranteed or endorsed by the publisher.

## Footnotes

^1^ http://www.ncbi.nlm.nih.gov/cdd

^2^ http://pfam.sanger.ac.uk

^3^ http://meme-suit.org

^4^ http://www.maizegenetic.net/bioinforFormatics/tasselindex.htm
